# Sialidases derived from Gardnerella vaginalis remodel the sperm glycocalyx and impair sperm function

**DOI:** 10.1101/2025.02.01.636076

**Published:** 2025-02-05

**Authors:** Sarah Dohadwala, Purna Shah, Maura Farrell, Joseph Politch, Jai Marathe, Catherine E Costello, Deborah J Anderson

**Affiliations:** 1 Boston University Chobanian and Avedisian School of Medicine, Boston MA; 2 Boston University, Boston MA

**Keywords:** bacterial vaginosis, sialidase, reproductive immunology, sperm, complement, Biological Sciences, Immunology and Inflammation

## Abstract

Bacterial vaginosis (BV), a dysbiosis of the vaginal microbiome, affects approximately 29 percent of women worldwide (up to 50% in some regions) and is associated with several adverse health outcomes including preterm birth and increased incidence of sexually transmitted infection (STI). BV-associated bacteria, such as *Gardnerella vaginalis* and *Prevotella timonensis,* damage the vaginal mucosa through the activity of sialidase enzymes that remodel the epithelial glycocalyx and degrade mucin glycoproteins. This damage creates an inflammatory environment which likely contributes to adverse health outcomes. However, whether BV-associated glycolytic enzymes also damage sperm during their transit through the reproductive tract has not yet been determined. Here, we show that sialidase-mediated glycocalyx remodeling of human sperm increases sperm susceptibility to damage within the female reproductive tract. In particular, we report that desialylated human sperm demonstrate increased susceptibility to complement lysis and agglutination, as well as decreased sperm transit through cervical mucus. Our results demonstrate a mechanism by which BV-associated sialidases may affect sperm survival and function and potentially contribute to adverse reproductive outcomes such as preterm birth and infertility.

## Introduction

Bacterial vaginosis (BV) is a condition characterized by vaginal dysbiosis that affects approximately one third of women globally (Peebles et al. 2019). In some regions in the global South, up to 50% of women have BV (Kenyon et al. 2013). This condition is highly associated with spontaneous preterm birth, increased risk of preclinical pregnancy loss, and STI acquisition (Howe et al. 1999; Ralph et al. 1999; Cauci and Culhane 2011; van Oostrum et al. 2013; Armstrong and Kaul 2021; Abou Chacra et al. 2023; Mohanty et al. 2023). In addition, emerging evidence suggests a link to infertility and pre-eclampsia, as well as adverse neonatal outcomes even for full term infants (Denney and Culhane 2009; van Oostrum et al. 2013; Dingens et al. 2016; Kindschuh et al. 2024). BV is an extremely variable condition, characterized by high diversity in the vaginal microbiome, and clinical symptoms such as irritation, discharge, itching, and fishy odor (Abou Chacra et al. 2022). To more precisely define the composition of vaginal microbiota found in humans, the field has adopted community state types numbered I to V to describe various proportions of different bacterial taxa (Ravel et al. 2011). A healthy vaginal microbiome, or CST I, consists primarily of *lactobacillus crispatus,* which maintains low vaginal pH and inflammation. A microbiome dominated by *lactobacillus iners*, termed CST II, is thought to be an intermediary microbiome between a healthy and disease state (Ravel et al. 2011). Dysbiotic vaginal microbiomes include other bacterial species such as *Gardnerella vaginalis* and *Prevotella Timonensis*, defined by CST III and IV.

To date, the mechanisms by which BV increases risk of adverse reproductive outcomes are poorly understood (Ugwumadu 2002; Mania-Pramanik et al. 2009l Elovitz et al. 2019; Gudnadottir et al. 2022); several have been proposed, including high vaginal pH, an inflammatory vaginal milieu, and BV-associated epithelial damage (Cauci et al. 2008). Recent findings have implicated glycolytic enzymes in remodeling the host vaginal glycocalyx (Agarwal et al. 2023; Segui-Perez et al. 2024). In particular, sialidases are often present in high concentration in clinical BV isolates and remove terminal sialic acids from glycoconjugates that decorate cell membranes. Endogenous sialidases are also present in the female reproductive tract (Flori et al. 2007; Ma et al. 2012). A key difference between endogenous and bacterial sialidases is that targeted host sialidases trim specific glycans during normal processes such as sperm capacitation, while in BV, promiscuous bacterial sialidases indiscriminately cleave host glycans important for reproductive and immune functions. BV-associated sialidases may forage nutrients from the vaginal tract mucosa that are required for BV associated bacteria and STI pathogens to thrive (Agarwal and Lewis 2021). A recent study demonstrated that sialylation of the vaginal epithelium was markedly lower in women with BV than those with a healthy microbiome, and that this phenomenon could be replicated ex vivo with a recombinantly expressed BV sialidase, NanH2 (Agarwal et al. 2023). Several other groups have corroborated this finding. One study showed that similar enzymes from another BV-associated bacterium, Prevotella, also removed sialic acid from the surface of vaginal epithelial cells; another demonstrated similar hydrolytic damage to mucins and other components of the vaginal mucosa (Pelayo et al. 2024; Segui-Perez et al. 2024).

Much evidence suggests that BV-associated damage to the vaginal mucosa could contribute to clinical symptoms of BV (Roselletti et al. 2020; Cheu et al. 2022). However, the mechanistic connection between BV associated changes to the vaginal environment and adverse reproductive outcomes, which primarily relate to the upper FRT, remains unclear. Thus, we have studied the effects of BV sialidases on spermatozoa, cells that transit through the vaginal environment and exercise important functions in the endocervix, endometrium and fallopian tubes (Mahdavinezhad et al. 2022). Their glycocalyx protects them from the female immune system, facilitates their passage through cervical mucus, and prevents premature capacitation, a process necessary for fertilization (Tecle and Gagneux 2015). The sialic acid-capped glycocalyx, termed the sialome, is particularly important (Fliniaux et al. 2022). Sialic acids are negatively charged moieties that cap glycan chains on the cell surface and bind several activating and immunosuppressive receptors in the Siglec family (Fliniaux et al. 2022). As a result, they are often part of a cell surface self-signal to prevent aberrant innate immune damage. Thus, sialic acid-mediated immune signaling within the female reproductive tract is thought to be important for the timely and appropriate induction of processes in fertilization and early pregnancy (Ma et al. 2012). In particular, the negatively charged sialic acid moiety allows sperm to navigate quickly through negatively charged cervical mucins and potentially bind to Siglecs on the endometrium and fallopian tubes to form sperm reservoirs prior to ovulation. Sialic acids may also mask highly immunogenic glycan epitopes on the sperm surface that are exposed during ovulation-induced capacitation and participate in fertilization(Ma et al. 2012). Thus, BV-associated sialidases may adversely affect sperm migration, immune signaling, and fertilization capacity.

The purpose of this study was to determine whether sialidases derived from *Gardnerella vaginalis* remodel the human sperm glycocalyx, prematurely exposing cryptic glycan epitopes and decreasing the negative surface charge of sperm. We used two approaches, lectin flow cytometry and zeta potential, to characterize the changes to the remodeled sperm glycocalyx after treatment with recombinant NanH2, a sialidase derived from clinically relevant strains of Gardnerella vaginalis that cleaves both *N-* and *O*-linked glycans (Agarwal et al. 2023). We next investigated the effect of desialylation on sperm motility through cervical mucus and sperm agglutination, and on innate immunity by investigating susceptibility of desialylated sperm to complement-mediated cytotoxicity. These data provide strong evidence that BV-associated sialidases can adversely affect sperm functions in the female reproductive tract, potentially contributing to the adverse reproductive outcomes such as preterm birth or subfertility that are associated with bacterial vaginosis.

## Results

### Sialic acids are depleted from the sperm glycocalyx after exposure to recombinant BV sialidase and bacterial supernatants, exposing cryptic epitopes and reducing negative surface charge.

**Figure 1:**
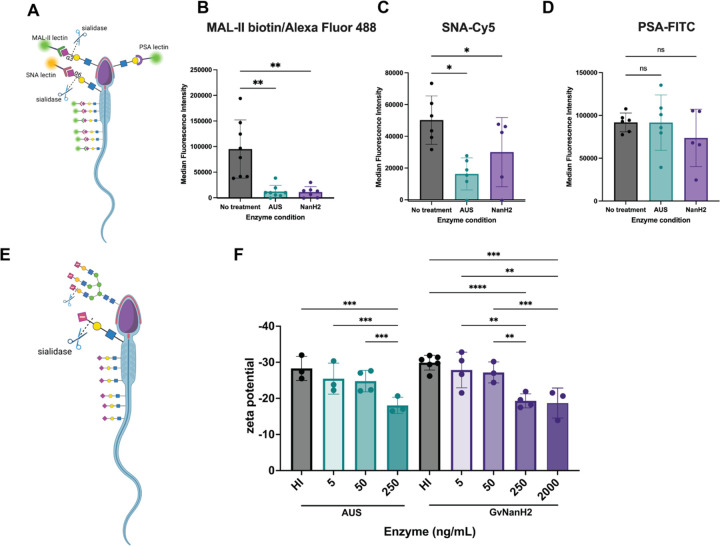
BV-associated sialidases remove surface sialic acid from sperm. A) Schematic describing lectin flow cytometry and representative glycan epitopes. B, C, D) Sperm were exposed to sialidases (AUS 20 units or GvNanH2 160 units) for 1 hour, fixed in formalin, and stained with MAL-II-biotin/Neutravidin-Alexa Fluor 488, SNA-Cy5, or PSA-FITC respectively. Each point represents the median fluorescence intensity from one donor. Statistics represent a mixed model comparison with post-hoc Holm- Šidák tests. E) Schematic representing change in zeta potential after removal of negatively charged sialic acid on sperm surface. F) Sperm were treated with sialidase at various doses for 1 hour, and zeta potential was measured via dynamic light scattering. Each measurement represents the average of seven technical replicates, and statistics represent a mixed model comparison with post hoc Holm- Šidák tests.

Sperm are rich in sialylated cell surface proteins that may be susceptible to hydrolysis by BV-associated sialidases. Accordingly, to investigate BV-associated damage to sperm, we produced recombinant, endotoxin-free GvNanH2 as described previously (Robinson et al. 2019). GvNanH2 is a truncated variant of a sialidase derived from Gardnerella vaginalis. To ensure that our experiments employed physiologically relevant sialidase activity, we used a reported value of enzyme activity derived from cervicovaginal swabs obtained from women with BV (0.5 micromoles/min 4U-MU hydrolysis, after accounting for dilution factors). In addition, we back-calculated amounts of active enzyme from specific activity values of the recombinant enzyme, and used BV-diagnostic values of 250 ng/mL and 2000 ng/mL of enzyme (Agarwal et al. 2023). Next, to confirm that BV-associated sialidases desialylated sperm-surface glycans, sperm were collected from healthy, reproductive aged men, isolated by density gradient, and treated with either recombinant GvNanH2, AUS (a commercially available sialidase derived from *A. urufaciens*, positive control), or no treatment (negative control). Then, we assessed removal of terminal sialic acids with lectin flow cytometry using SNA-Cy5 and MAL II-biotin to measure a decrease in 2, 6 and 2,3-linked terminal sialic acids, respectively (Fig 1A). The untreated controls demonstrated the presence of 2, 3 and 2, 6 linked sialic acids on the sperm surface (Fig 1B, 1C). This corresponds to results from previous studies using lectin microarrays to profile the sperm surface glycome (Xin et al. 2014). For SNA-Cy5 staining, the direct conjugate enabled a direct comparison of the activity of AUS and GvNanH2. AUS reduced the signal by roughly 50% relative to the control, while GvNanH2 reduced it by approximately 15% (Fig 1C). Similarly, a significant reduction was observed in the staining for MAL-II, though due to a secondary amplification step, a linear relationship to compare AUS and GvNanH2 was not possible. Although not statistically significant, a three-fold decrease in signal was observed with NanH2 compared to AUS (Fig 1B).

Sperm, being foreign to the female reproductive tract, have immunogenic epitopes capped with terminal sialic acids that function in processes such as the acrosome reaction and sperm-egg interaction. To determine if the glycan moieties underlying terminal sialic acids were exposed or degraded after sialidase treatment, we used lectin flow cytometry with pea savitum agglutinin (PSA), a lectin which binds *N*-acetyllactosamine residues and is a marker for the acrosome reaction (Fig 1A). In untreated (control) sperm, robust PSA staining was observed (Fig 1D). In both sialidase treatment conditions (AUS and NanH2), sperm PSA staining was unchanged from that of the control (Fig 1D). This indicates that sialidase treatment does not degrade other glycan moieties nonspecifically and does not result in a premature acrosome reaction.

The sperm sialome contributes to a negative surface charge. To determine if the surface charge of sperm is modified in response to sialidase treatment, the zeta potential of sperm cultures was measured in response to different doses of enzyme. First, we optimized the diluent for the assay and confirmed the effect of different culture conditions on the measurement (Fig S4A). A decrease in sperm surface charge was observed when whole semen was treated, though the magnitude of the change observed was lower than when washed sperm were treated. It is possible that sialylated glycoproteins in seminal plasma protect the sperm surface from the full force of the sialidase enzymes through a competition mechanism. Next, we measured the change in zeta potential to compare the same doses of recombinant NanH2 and AUS and found that effects were similar (Fig S4C). Finally, we used a physiological relevant series of doses of both NanH2 and AUS sialidase and measured the zeta potential of the cultures. We observed a dose dependent decrease in the magnitude of the negative surface charge after a 30 min incubation for both AUS and NanH2 (Fig 1F). We also measured the donor-to-donor variance in the biological replicates, and found that there was some variance in how the sperm from different donors were affected by the sialidases, though overall trends in the data were retained (Fig S4D).

### Desialylated sperm are more susceptible to spontaneous, antibody- and lectin-mediated agglutination

Agglutination is a phenomenon driven by cell motility and surface charge wherein motile sperm collide and stick to one another, forming large, aggregated networks. We investigated the propensity of highly motile sperm to agglutinate after sialidase treatment. We hypothesized that by reducing the negative charge, sperm desialylation would make the sperm stickier and prone to agglutination.

To measure this, washed sperm were treated with different doses of AUS sialidase and observed over time. We observed variable degrees of sperm agglutination under these conditions. The variable characteristics in these spontaneous agglutinates included time to agglutination and size and shape of agglutinates. Next, we tested whether sperm desialylation affects antibody or lectin-induced sperm agglutination. To quantify this effect, we performed a kinetic agglutination assay on sperm treated with sialidase or heat inactivated enzyme (negative control), followed by Galectin-1, the Human Contraception Antibody (HCA), or Pea savitum agglutinum (PSA). These entities were selected due to their importance in reproductive biology. Galectin-1 is involved in binding STI pathogens and is expressed in FRT tissues. HCA, derived from the serum of an infertility patient, is part of a contraceptive product that is poised to enter Phase II clinical trials (Anderson et al. 2020). Finally, PSA is a marker for the acrosome reaction, a process crucial to sperm-egg interaction. These three entities bind distinct glycans-- HCA binds a male reproductive tract specific, but largely uncharacterized sperm *N*-glycoform; Galectin-1 binds repeat *N*-acetyllactosamine units, and PSA binds terminal mannose residues. None of these three entities bind sialic acid moieties specifically, though they may all be tolerant of sialylated glycans.

**Figure 2:**
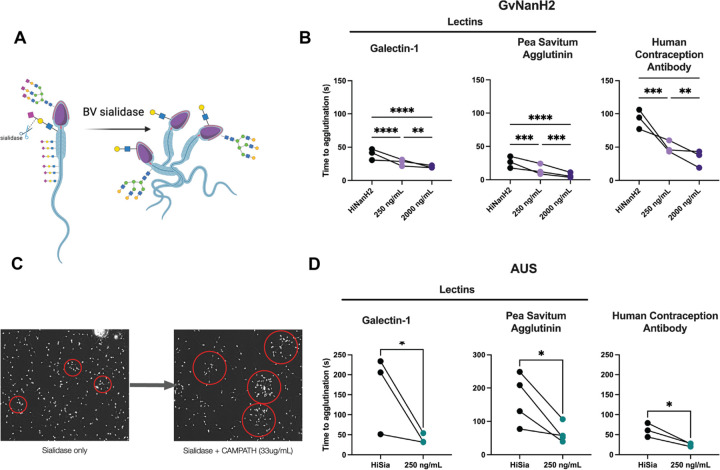
Desialylation results in faster sperm agglutination. A) Schematic representing sperm agglutination after sialidase treatment. C) Representative image of spontaneous, incomplete sperm agglutination after sialidase exposure, and incomplete agglutination after sialidase and CAMPATH exposure. Agglutinates are circled in red. B, D) Time to agglutination in the presence of different lectins and glycan-binding antibodies after treatment with GvNanH2 or AUS, respectively. Treatment with heat inactivated (HI) NanH2 or AUS (250ng/ml) was included as a negative control. Statistics represent repeated measures ANOVA with post hoc Holm-Šidák tests for NanH2 (1B) and unpaired t-tests for AUS (1C).

We optimized the concentrations of the agglutinating antibodies and lectins, selecting low concentrations that were weakly agglutinating to distinguish the effect of sialidases treatment. We found that sperm agglutinate significantly faster when exposed to each of the three agglutinating entities after treatment with either AUS or NanH2 sialidase (Fig 2B, 2C). In addition, for PSA, flow cytometry data indicated that surface staining is unchanged in response to sialidase treatment, suggesting charge-mediated repulsion in preventing agglutination, rather than enhanced binding of underlying glycan epitopes in treated sperm. In sum, removal of surface sialic acids from sperm increased their propensity to agglutinate spontaneously and in the presence of several distinct lectins and glycan-binding antibodies.

### Desialylated sperm are more susceptible to complement-mediated immobilization and cytolysis

Sialic acids prevent aberrant complement activation by binding several complement inhibitors including Factor H (Blaum et al. 2015). Both complement and complement inhibitors can be found in human cervical mucus and seminal plasma (Price and Boettcher 1979; Petersen et al. 1980) suggesting that complement biology may be important to fertility and reproduction, though specific mechanisms are not yet fully understood. For instance, complement proteins may serve as a bridge between sperm and egg (Anderson et al. 1993), and some antisperm antibodies demonstrate potent complement-dependent immobilization and cytolysis of sperm (Baldeon-Vaca et al. 2021).

**Figure 3:**
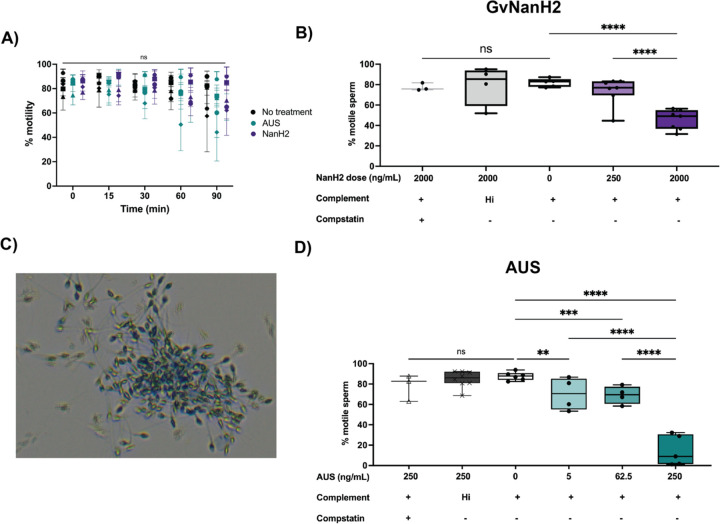
Effect of sialidase treatment on sperm motility and susceptibility to complement-mediated immobilization. A) Sperm motility was measured after sialidase treatment (2000ng/mL NanH2 and 250 ng/mL AUS) via computer assisted sperm analysis (CASA). Statistics represent a repeated measures comparison; neither time nor enzyme condition was a significant source of variation. B) Sperm motility was measured after treatment with GvNanH2 sialidase in the presence of complement. Heat-inactivated complement and 125 uM compstatin (a human C3 inhibitor) were used as negative controls. C) A representative image of a sperm agglutinate after treatment with NanH2 sialidase and complement for 1 hour. Sperm were stained with trypan blue to detect cell death. Dead sperm were observed, along with motile live sperm (sometimes seen as tracks in the images due to movement). D) Same as B), but with AUS sialidase. Statistics represent mixed model comparisons with post-hoc Tukey tests.

We used the complement-mediated sperm immobilization assay to determine the potential role of sialidase enzymes in spontaneous- and lectin- mediated complement activation. We first investigated whether sialidase treatment alone affected sperm motility. We did not observe a significant effect of either NanH2 or AUS on sperm motility through 60 minutes, after which time we observed a slight decrease in sperm motility in some donors (Fig 3A). Next, we examined the effect of sialidases on sperm motility in the presence of complement. Addition of 2000 ng/mL GvNanH2 with complement significantly reduced sperm motility compared to all three negative controls (no enzyme treatment, heat inactivated complement, addition of compstatin, a C3 complement inhibitor) (Fig 3A). We also used AUS sialidase to examine how additional sialic acid linkages may play a role. In comparison to AUS, GvNanH2 is reported to cleave α 2–3, 6 linkages; its activity against α 2–8,9 linked sialic acids is unconfirmed. We observed that treatment with much lower doses of AUS sialidase resulted in substantial reductions in sperm motility in the presence of complement, indicating a strong role for α 2–8,9 linked sialic acids, primarily found in poly-sialic chains, in protection from complement lysis (Fig 3B). We also tested a lower dose of the complement inhibitor compstatin (25 μM) to determine if alternative complement activity accounted for sperm immobilization, since compstatin has a lower IC_50_ (12 μM) to inhibit the alternative pathway. Complement activity was hindered, but not completely abrogated (Fig S6). This indicates that this complement activity likely occurs through the lectin pathway rather than the alternative pathway. Finally we confirmed that complement-mediated sperm immobilization is a surrogate for cell lysis (Fig 3B). After complement treatment of sialidase-treated sperm the majority of immotile sperm stained positive for trypan blue, indicating that they were lysed. The majority of lysed sperm were present in agglutinates, which could indicate that sperm become sticky after lysis, or that agglutination facilitates complement activation on the surface of sperm due to increased size (Dalia et al 2011). In addition, we explored antibody-mediated complement lysis, and sperm were more prone to complement lysis through the classical complement pathway in the presence of sialidase (Fig S5). In sum, these data provide evidence that surface sialic acid moieties protect sperm from complement-mediated lysis, likely through lectin mediated pathways.

### Desialylated sperm are less able to transit through human cervical mucus

**Figure 4:**
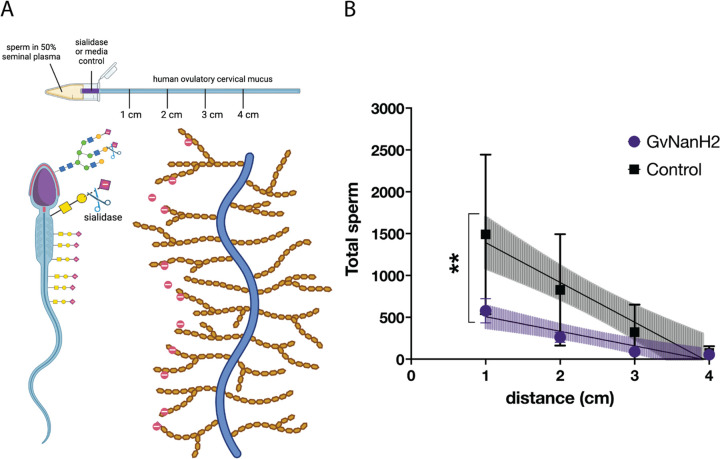
Sperm transit through cervical mucus is reduced after desialylation. A) Schematic depicting experimental setup and mucus-sperm interaction model. Washed motile sperm were incubated with seminal plasma, then inserted into a capillary tube containing sialidase or media at the entrance and ovulatory cervical mucus B) After 90 minutes, sperm were quantified at 1-cm intervals in the capillary tube. Statistics represent an unpaired test comparison of the slopes of the lines derived from the four points. Data represent three biological replicates with either two or three technical replicates depending on donor volume.

Human cervical mucus varies in physical properties and composition throughout the menstrual cycle and is rich in negatively charged mucins. Sperm must transit across this mucosal barrier to access the upper reproductive tract and fertilize an egg. To determine how sperm transit through mucus may be affected by sialidase treatment, we measured numbers of sperm observed at progressive distances through a capillary tube filled with ovulatory human cervical mucus. We started with a model that simulates sperm transit through the vagina and cervix after intercourse (Fig 4A). Motile sperm were isolated, resuspended at a concentration of 80 million/mL in 50% seminal plasma, and placed into a capillary tube to measure migration through a small region (0.5 cm) of GvNanH2 sialidase or media (control), followed by midcycle cervical mucus (Fig 4A). We found a significant reduction in the numbers of total sperm that were able to transit through the mucus after exposure to sialidase (Fig 4B).

Next, we measured different methods of sialidase exposure since the localization of the bacterial enzyme in the cervix is unknown. We mixed human midcycle cervical mucus with recombinant sialidase (amount equivalent to 0.5 μM/min 4-MU hydrolysis activity) or media (control) and counted the number of sperm that penetrated to various depths of the capillary tube after 90 minutes. Pre-treatment of mucus with sialidase initially facilitated sperm transit, but the sperm became immotile as they traversed the sialidase-containing mucus (Fig S8). While the numbers of sperm found in mucus increased in the sialidase-containing mucus, the percent motile sperm decreased, which was the opposite of the trend observed in untreated mucus (Fig S8). This may be due to more efficient complement lysis of the sperm exposed to sialidase during transit through the cervical mucus. Next, we tested treatment of sperm with sialidase prior to mucus exposure and found that this also reduced their transit through cervical mucus (Fig S8). We also tested these phenomena with and without seminal plasma, which contains several proteins known to coat the sperm surface (Fig S7). We found that seminal plasma protects sperm from the immobilization observed in sialidase-containing mucin, perhaps due to abundant seminal sialoglycoproteins in seminal plasma (Fig S7). Finally, we also compared sperm transit through human cervical mucus to a commonly used commercially available mucin model, bovine submaxillary mucin. In the absence of seminal plasma, a similar pattern was observed; however, in the presence of seminal plasma, human cervical mucus was much more permissive to sperm transit (data not shown). This indicates some specific interaction between components of human cervical mucin and seminal plasma that is not be recapitulated with bovine mucin. In sum, our data indicate that BV-associated sialidases affect sperm transit through human midcycle cervical mucus, both by altering the sperm’s forward progression in mucus, and by altering the properties of the mucus itself.

## Discussion

Our study demonstrates that that BV-derived sialidases alter the sperm glycocalyx by removing terminal sialic acid residues, and provides evidence that the unperturbed sperm glycocalyx likely protects sperm from immune effectors present in the female reproductive tract and facilitates their transit through cervical mucus. Desialylated sperm were highly susceptible to complement-mediated damage and agglutination, and penetrated midcycle cervical mucus poorly. Prior to our work, most studies on the effects of BV-associated sialidases were limited to the vaginal mucosa (Agarwal et al. 2023; Segui-Perez et al. 2024), and the work of groups looking at the importance of the sperm sialome in bacterial infection and capacitation (Khosravi et al. 2016). Studies related to the effects of sialidases on sperm were limited in humans, though some animal models supported the idea that exposure to sialidases affects sperm function in the reproductive tract (Ma et al. 2016; Fernandez-Fuertes et al. 2018).

Several orthogonal lines of evidence support the idea that BV-associated sialidases may damage sperm and affect fertility: 1) *G.vaginalis* is found at a higher frequency in semen from infertile men (Andrade-Rocha 2009) and oligozoospermic seminal plasma contains higher levels of free sialic acid relative to normozoospermic samples (Menevse et al. 2022), 2) other bacterial conditions that involve glycolytic enzymes are mechanistically linked to male infertility (Khosravi et al. 2016), 3) multiple studies with sperm from various other mammalian species have pointed to the importance of the glycocalyx in protecting from innate immunity in the FRT (Fliniaux et al. 2022), 4) the semen microbiome is associated with sperm health (He et al. 2024; Osadchiy et al. 2024) and 5) natural reproductive processes, such as capacitation, the acrosome reaction and sperm-egg fusion, are sialidase-dependent and rely on the timely exposure of relevant surface glycans (Feng et al. 2016). Finally, several animal models demonstrate that sialic acid moieties on sperm can impact immune cells and endometrial reproductive processes (Toshimori et al. 1991; Froman and Thursam 1994; Machado et al. 2014; Álvarez-Rodríguez et al. 2020; Batra et al. 2020; Schjenken et al. 2021; Donnellan et al. 2023; Li et al. 2024). In sum, human sperm are an underappreciated target for bacterial sialidase enzymes associated with BV, and premature desialylation likely impacts sperm function and the immunology of the female reproductive tract.

We found that desialylated sperm provoke complement activation via the lectin-mediated pathway. Prior work has demonstrated that semen and sperm mediate inflammation in the lower reproductive tract in the absence of BV-associated bacteria, and thus it is possible that BV-associated damage to sperm exacerbates this existing inflammatory effect through pathways such as excessive complement activation (Schjenken et al. 2021). Higher levels of complement components C3 and C5, and mediators such as mannose binding lectin have been found in women with BV who deliver preterm, and so it is possible that sperm are doubly susceptible to damage from the FRT immune system in the BV condition-- first via desialylation, and second via the increased levels of innate immune mediators in the cervicovaginal environment (Chan et al. 2022). Given that women with BV are already at a heightened state of immune activation at baseline, desialylated sperm may kickstart an ascending immune response. Despite the lack of a definitive mechanism for the association between preterm birth and BV, the field has coalesced on an ascending infection/inflammation model to explain aberrant inflammatory processes in the upper reproductive tract (Tantengco and Menon 2022). These aberrant processes in early pregnancy may result in poor decidualization that drives adverse outcomes such as preterm birth (Norwitz et al. 2001; Norwitz 2006). Given that sperm transit through the lower reproductive tract to the upper tract, sustaining immune damage and activating complement throughout their route, we hypothesize that sperm may initiate and carry some of these aberrant immune activation cascades into the upper tract prior to fertilization. Initial bursts of inflammation are required for processes in early pregnancy such as implantation and placentation, but overactivation of these pathways results in pathology; perhaps desialylated sperm are partially responsible for the imbalance.

Next, we found that desialylated sperm are more prone to agglutination, probably due to a reduction in the surface charge that removes the inherent cell-cell repulsion that sperm use to avoid attachment to other sperm. Enhanced agglutination may mean that contraceptive antibodies and lectins will be more efficacious in women with sialidase-positive BV, but also that sperm may be more prone to agglutination in the absence of antibody as well(Tsuji et al. 1988; Baldeon-Vaca et al. 2021). Given that several leading clinical contraceptive candidate antibodies, as well as a few anti-HIV antibodies, rely on glycan binding, it will be important to investigate how women with sialidase-positive BV respond to these topical glycan-binding drug products(Anderson et al. 2017). Furthermore, several groups have proposed that sperm may carry STI pathogens into the upper tract via cell-cell sticking, and vaginal sialidases may exacerbate this phenomenon (Young et al. 2019).

We found that desialylated sperm traverse less successfully through ovulatory cervical mucus. This supports rich glycosylation data from cervical mucus in the sheep model, where interesting differences in sialic acid content on the cervicovaginal tissue and mucus were found in sheep with impaired fertility (Abril-Parreño, Krogenæs, et al. 2022). Studies on the nature of ovulatory human cervical mucus are hindered by the difficulty of sample collection, particularly from women with untreated bacterial vaginosis and in the absence of hormonal birth control, which can modify mucus characteristics (Moncla et al. 2016). It is known that women with BV produce more mucus, perhaps to counteract the bacterial metabolism of mucin glycoproteins observed in BV (Moncla et al. 2016). However, it remains understudied whether sialidases remain in the vagina or ascend into the cervical environment and upper reproductive tract in women with sialidase-positive BV.

This work has some limitations. We did not collect samples from infertile or subfertile men, so we cannot make assertions about male infertility outside the FRT, or how bacterial sialidases may exacerbate pre-existing male infertility. In addition, we did not screen sperm donors for BV-associated bacteria, though sperm samples with poor motility were excluded. In addition to sialidases, several other glycolytic enzymes including amylases and fucosidases are produced by BV-associated bacteria that we have not yet explored. Finally, the diversity in bacterial vaginosis cases should also be further considered, given that molecular profiling has revealed varying presentations of vaginal flora (Savicheva et al. 2023). *Gardnerella vaginalis*, which produces the sialidase of focal interest in this study, is a primary cause of BV (Morrill et al. 2020). However, it should be noted that not all genotypes of G. *vaginalis* produce sialidase, and this may cause variation in clinical presentation of BV cases, and that other species such as Prevotella and mycoplasma, express sialidases (Hardy et al. 2017; Pelayo et al. 2024). Clearly, in adverse reproductive outcomes associated with BV, there is also a role of the damage to the FRT. This work does not study the interactions between damaged FRT and sperm. The *in vitro* environment may not recapitulate certain aspects of physiology, such as fluid flow, muscle contractions, as well as the menstrual cycle and hormone signaling on sperm.

In summary, we demonstrate that BV-associated sialidases increase sperm susceptibility to damage in the FRT, and that this may play an underappreciated role in adverse reproductive outcomes. Understanding aberrant immune damage to sperm in bacterial vaginosis raises new questions about how sperm may modify the immune milieu of the female reproductive tract in both a eubiotic and dysbiotic condition. These findings highlight the need for further investigation of the role of sperm in decidualization and ascending inflammation in the upper reproductive tract, as well as a better foundational understanding of important processes of early pregnancy in humans.

## Materials:

### Enzymes and substrates:

AUS Sialidase refers to Neuraminidase A (New England Biolabs P0722L). 4-MU Sialic acid refers to 2′-(4-methylumbelliferyl)-α-D-*N*-acetylneuraminic acid sodium salt hydrate (Sigma Aldrich 69587). GvNanH2 refers to truncated recombinant Gardnerella vaginalis derived NanH2, as described below.

### Bacterial strains, plasmids and DNA:

ClearColi bacteria strains that can produce endotoxin-free protein were a gift of the Dempsey lab at BUMC. The GvNanH2 plasmid was a gift of the Lewis lab at UCSD.

### Production of recombinant NanH2 (GvNanH2):

The NanH2 plasmid was acquired from the Lewis lab at UCSD (Lewis et al. Production of NanH2). The plasmid contains a functional, soluble, His-tagged, truncated form of NanH2, a sialidase expressed by *Gardnerella vaginalis*, a BV-associated species, and a gene encoding kanamycin resistance on a pET28a backbone. The plasmid was transfected into ClearColi by heat shock, then streaked for single colonies on kanamycin selective luria bertani (LB) agar plates. The identity of the plasmid was confirmed by whole plasmid sequencing (Plasmidsaurus). Colonies were picked after 2 days (ClearColi grows at half the rate of BL21 E.coli), and inoculated overnight at 37° C with shaking in a 10 mL LB-Kanamycin culture. Larger 250 mL cultures were inoculated and grown untill they reached optical density 0.4. Cultures were induced with 250 mM Isopropyl ß-D-1-thiogalactopyranoside (IPTG) and grown overnight at 37° C after which time the bacteria were pelleted, and stored in a lysis buffer (50 mM NaH2PO4 at pH 7.4, and 300 mM NaCl) at −80°C until use. To purify protein, pellets were thawed, mixed thoroughly with a serological pipette, and then lysed in a French pressure cell press thrice. The lysate was clarified by centrifugation thrice, then incubated with Nickel NTA resin while shaking at room temperature for one hour. The resin was pelleted and washed thrice in wash buffer (20 mM sodium phosphate, 300 mM sodium chloride, 25 mM imidazole), and then bound enzyme was eluted thrice in the elution buffer (20 mM sodium phosphate, 300 mM sodium chloride, 250 mM imidazole). The eluants were pooled, buffer exchanged (to remove imidazole) into storage buffer (50 mM NaCl, 20 mM Tris-HCl, 1 mM EDTA, pH 7.5), assessed for protein concentration via BCA, run on an SDS page gel, then aliquoted into single use aliquots stored at −80° C until use.

### Lectins and antibodies:

Biotinylated Maackia Amurensis Lectin II (MAL II, Cat #:B-1265-1) and Cy5-conjugated Sambucus Nigra Lectin (SNA, Cat #: CL-1305-1) were purchased from Vector Labs. Human Contraception Antibody (HCA), was a gift from ZabBio. Recombinant human Galectin-1 was purchased from ACROBiosystems (Cat# 50-210-6461) and Pea savitum agglutinin (PSA-FITC) was purchased from Sigma (cat #L0770).

## Methods:

### LC/MS-MS to confirm sequence of GvNanH2:

In-gel digestion using trypsin/Lys-C and LC-MS/MS were used to confirm the protein sequence of the recombinant enzyme. LC-MS/MS analysis was carried out using a nanoAcquity UPLC (Waters Technology) interfaced to a Q-Exactive HF mass spectrometer (ThermoFisher Scientific.) Reversed-phase C-18 analytical and trapping columns were used with a 75 minute method with a gradient of 2% B to 40% B in 40 minutes, using 99% water/1% acetonitrile/0.1% formic acid as mobile phase A and 99% acetonitrile/1% water/0.1% formic acid as mobile phase B at a flow rate of 0.5 μl/min. Data-dependent tandem MS spectra were acquired in positive mode for the top 20 most abundant precursors. Full MS scans were acquired from *m/z* 350 to 2000 with a resolution of 60,000 using an automatic gain control target of 3e6 and maximum injection time (IT) of 100 ms. Dynamic exclusion (12 s) was enabled. Precursor ions were fragmented using a resolution of 15,000 with a maximum injection time of 50 ms and an automatic gain control value of 2e5 using higher energy collision-induced dissociation with a stepped normalized collision energy of 27 and 35 V. The MS/MS spectra were searched using Byonic against a fasta file containing the truncated NanH2 sequence and provided assignments for 81 peptides; these represented 83.2% of the predicted sequence.

### Sperm isolation:

Semen was acquired from healthy donors after obtaining written informed consent with the approval of BUMC Institutional Review Board (IRB) under protocol #H36843, and processed as described previously in Mausser et al. In brief, samples were processed within one hour of acquisition; motile sperm were separated from whole semen on a 90% ISolate density gradient (FUJIFILM Irvine Scientific; Santa Ana, CA, USA) and centrifugation at 300g for 20 minutes. The motile sperm pellet was resuspended in Multipurpose Handling Medium (MHM; FUJIFILM Irvine Scientific; Santa Ana, CA, USA). Sperm concentration and motility of both whole semen and isolated motile sperm were assessed using a Computer-Assisted Sperm Analysis system (CASA; Human Motility II software, CEROS II, Hamilton Thorne, Beverly, MA, USA). Samples with low concentrations (<10 million/mL) or motility (<40%) post-processing were not used for agglutination and complement experiments.

### Sperm sialidase treatment:

After isolation of motile sperm, cells were resuspended to a concentration of 30 million cells per mL in multipurpose handling media (MHM) media. 40 μL reactions with 1 μL of enzyme were incubated at 37°C for the chosen time-points and enzyme concentration. All enzymes were thawed on ice, used fresh, and diluted into identical storage buffers (50 mM NaCl, 20 mM Tris-HCl, 1 mM EDTA, pH 7.5) to maintain identical media conditions and concentrations, even as enzyme concentration varied.

### Sialidase activity assay:

Activity was confirmed by 4MU hydrolysis assay as previously described (Robinson et al. 2019). In brief, to measure sialidase activity and match levels of AUS and NanH2 activity, a 4-methylumbelliferone-sialic acid assay was used. 20 μL of enzyme solution was diluted into 100 μL of 600 μM 4MU-sialic acid (Sigma Aldrich 69587), and immediately measured for fluorescence in a BioTek plate reader. The measurement was performed in a 96 well plate every 1 minute for 2 hours at 37°C (Ex/Em: 365nm/448nm, Gain=43). A standard curve of 2-fold dilutions in triplicate of 4MU starting at 150 μM and ending with 2.34 μM was used to convert arbitrary fluorescence units into μM 4MU hydrolysis per minute. The first 12 minutes of the reaction (<10% substrate consumption) was used as a linear portion of the curve to calculate activity. rNanH2 (produced recombinantly as described above) was used at a final concentration of at 0.27 ng/μL. AUS enzyme (NE BioLabs P0722L) at 20000 U/mL was diluted into 100 mM sodium acetate buffer (pH 5.5) for a final dilution of 1.8U/μL. Enzymes were thawed on ice until use.

### Inhibition of Sialidase Activity Assay:

To measure activity of inhibited sialidase enzyme, activity assays were performed using purified NanH2 enzyme and N-Acetyl-2,3-dehydro-2-deoxyneuraminic acid inhibitor (DANA, Sigma Aldrich D9050). rNanH2 was used at a final concentration of at 0.27 ng/μL. DANA was used at final concentrations ranging from 1 mM to 10 pM. Enzyme and inhibitor were added in triplicate in a black-bottom 96 well plate. The substrate, 4-methylumbelliferone-sialic acid (Sigma), was added immediately prior to reading the plate. Fluorescence activity was measured in a BioTek plate reader every 1 minute for 2 hours at 37°C (Ex/Em: 365nm/448nm, Gain=43). A standard curve of 2-fold dilutions in triplicate of 4MU starting at 150 μM and ending with 2.34 μM was used to convert arbitrary fluorescence units into μM 4MU.

### Lectin flow cytometry:

To measure staining of sperm with lectins, sperm were incubated with 160 U NanH2, 20 U AUS, or no treatment/heat-inactivated enzyme for 1 hour and were fixed in formaldehyde prior to staining. After fixation, 5×10^4^ sperm were stained per condition at a final lectin concentration of 10 μg/mL for primary conjugates and 5 ug/mL for biotin conjugates for 30 minutes. Biotin conjugates were stained with Neutravidin-Alexa Fluor 488 at 2 μg/mL for 45 minutes. Sperm were analyzed using a Cytek Aurora full spectral flow cytometer. Cells were gated by forward and side scatter and doublets were removed by gating the linear FSC-A vs FSC-H. Populations were compared by determining median fluorescence intensity for each condition in FlowJo software.

### Zeta potential measurements:

Zeta potential was measured by diluting 20 μL of sperm at a concentration of 30 million per mL into 1 mL of distilled water right before measurement. A Malvern zetasizer instrument was used at the following settings. The diluent setting selected was 10mM NaCl, and seven measurements were taken per sample with no cooldown time between measurements.

### Sperm agglutination assay:

Sperm were treated with selected levels of sialidase as described. Then, a kinetic agglutination assay was performed as described in prior work, to measure time to 95% agglutination with HCA, Galectin-1 and Pisum sativum agglutinin (PSA (LALA paper).

### Complement mediated sperm immobilization:

Sperm were treated with sialidase as described, then exposed to human serum complement and selected concentrations of antibody for 1 hour. Assay was performed as previously described (LALA-PG paper). In brief, motile sperm were quantified with CASA software after complement exposure. Heat inactivated sialidase enzyme and complement were used as negative controls. A human complement inhibitor was used as an additional negative control. Serum complement and compstatin (C3 inhibitor) (MedChemExpress, USA) were incubated together for 15 mins at room temperature and then sialidase treated sperm were added as previously described. Non-classical complement mediated sperm immobilization was measured with an identical protocol, but using MHM media instead of antibody.

### Cervical mucus penetration assay:

Cervical mucus samples were acquired after obtaining written informed consent with the approval of BUMC Institutional Review Board (IRB) under protocol #H41454. Midcycle ovulatory cervical mucus was collected from reproductively aged women using an endocervical pipelle (Aspirette Endocervical Pipelle, Cooper Surgical, Trumbull, CT, USA) within 48 hours of a positive ovulation test (Digital Ovulation Predictor Kit, Clearblue). Participants had regular menstrual cycles and were not taking any hormonal birth control. The samples were stored at 4 C until use (within 5 days). Cervical mucus was diluted at a 1:3 ratio of mucus: PBS and aspirated into capillary tubes (Borosilicate Capillary Glass Slide, 0.30×3.0 mm, 50 mm, Electron Microscopy Sciences, Hatfield, PA, USA) using a squirrel feeder and syringe. One end of the tube was sealed using nail enamel, another end was filled with 10 μl of multipurpose handling media (MHM) containing NanH2 sialidase or media alone as a control. The capillary tube was inserted horizontally in a 1.5ml microcentrifuge tube containing 80 μL of 20 ×10^6/mL motile sperm. The assay was performed as previously reported by Mausser et al. CASA images were taken at different depths in the capillary tube to count motile sperm for each distance in the capillary.

### Sperm motility assay:

Sperm motility over time was quantified using CASA software and specialized microscope slides with a shallow chamber depth. Sperm at 30 million per mL were treated with different sialidases, incubated at 37C in a wet box, and measurements were taken at specific time points in the CASA. At least 600 sperm were measured per condition to determine a percent motility in the CASA, corresponding to >3 fields of view per time point per condition.

### Sperm Live/Dead staining after complement treatment:

Sperm were incubated with sialidase and complement, as described in the complement-mediated immobilization assay. Motility was measured with the CASA, then cells were stained with trypan blue (1:1) to confirm whether percent motility matched with the percentage of dead cells (trypan positive).

### Statistical analysis:

Continuous variables with >2 conditions were analyzed by analysis of variance (ANOVA) or mixed-model analysis. A significant effect was followed by Holms-Sidak multiple-comparison tests, or a Tukey multiple-comparison test when Holms-Sidak could not be calculated. Variables were tested for normality by the Shapiro-Wilk test. If continuous variables were not normally distributed, they were log (natural)- transformed prior to analysis. For repeated measures ANOVA, the equality of variances of the pairwise differences between within-subject conditions was assessed through tests of sphericity (Greenhouse-Geisser epsilon and Huynh-Feldt epsilon). For 2-group/condition comparisons of continuous variables, unpaired or paired t-tests or Mann-Whitney U tests or Wilcoxon matched-pairs signed-rank tests comparing the slopes of linear regression lines were performed. Statistical significance was assumed when P<.05. Data analysis and graphing were performed with Prism, Version 9.4.1 (GraphPad Software, Inc, La Jolla, CA) software.

## Supplementary Material

Supplement 1

## Significance statement

Sperm surface glycans are crucial for reproductive and immune processes in the female reproductive tract. We found that sialidases derived from BV-associated bacteria remodel the sperm glycocalyx resulting in aberrant agglutination, mucus trapping, and complement-mediated cytolysis of sperm. These results implicate glycolytic damage to sperm as a potential mechanism for BV-associated adverse reproductive outcomes.
