# Supplementary material for: Sialidases derived from Gardnerella vaginalis and Prevotella Timonensis remodel the sperm glycocalyx and impair sperm function
Source: bioRxiv. 2025 May 29:2025.02.01.636076. Originally published 2025 Feb 5. Preprint. [Version 2] doi: 10.1101/2025.02.01.636076 (PMC11838519; doi:10.1101/2025.02.01.636076)
Supplement: Supplementary file 1 [file media-1.docx]

A_ 

B

C 

D 

**Supplementary Figure 1: Production of GvNanH2.** A) SDS PAGE of purified GvNanH2. B) Activity determination of different concentrations of GvNanH2 with 4-MU hydrolysis assay. N= 5 C) Activity comparison to 5 units of commercially available AUS sialidase. N= 5  D) Inhibition of GvNanH2 by DANA, a sialidase inhibitor. 95% confidence intervals are represented by the pink dashed line. N = 3. Error bars represent standard error of the mean.  

**Supplementary** **Figure** **2**: **LC-MS/MS- based sequence coverage of in-gel digest confirms sequence of GvNanH2.** The 75 Kda band from the SDS PAGE gel in Fig S1A was cut and digested with trypsin, and peptides were subjected to LC-MS/MS analysis to confirm the identity of the protein. 

**Supplementary Figure 3: Flow cytometry gates for sperm.** Sperm were fixed in formalin, stained with MAL-II, and analyzed by flow cytometry. Gates were selected to select sperm and remove doublets based on forward and side scatter. 

**Supplementary** **Figure** **4:** **Zeta** **potential** **method** **development**. A) Diluent optimization of reducing variability within technical replicates. B) Zeta potential and sperm culture conditions. C) Comparison of  AUS (green) and NanH2 (purple). D) Biological replicates shown individually to show technical replicates within each sample, and highlight donor to donor variability. 

**Supplementary Figure 5: ZB06 sub-analysis indicates a nonsignificant correlation between increased immotile sperm counts and higher Nugent score in the absence of HCA film and a decrease in the presence of HCA film.**  Immotile sperm counts derived from post-coital test were correlated to Nugent scores from participants using a Pearson correlation matrix. The Nugent scores for participants were collected in separate clinic visits from the post-coital test visits. 

**Supplementary Figure 6: Human Contraception Antibody (HCA) mediated complement immobilization of sperm is enhanced in the presence of sialidase.** AUS sialidase at 20 units was incubated with sperm for 1 hour, then complement was added and incubated for 1 hour. Motility of these samples was quantified by CASA software. Each point represents the average of biological replicates and error bars represent standard deviations.  A mixed effects analysis revealed that there was a statistically significant effect on motility due to the sialidase treatment and antibody dose (p<0.0001), and post-hoc Holms-Sidak testing revealed a difference in the sialidase treatment conditions in the absence of antibody (p=0.0033). N = 5 to 7 biological replicates for the complement conditions, and N = 2 for the heat inactivated complement conditions (HiC). 

**Supplementary Figure 7: Compstatin dose range in complement immobilization assay. Statistics** represent a mixed model comparison with post-hoc Holms-Sidak comparison.

**Supplementary Figure 8: Effect of seminal plasma on cervical mucus transit of sperm.** A cervical mucus transit assay was performed with one cervical mucus sample containing sialidase enzyme in the presence and absence of seminal plasma. The bubble size represents total sperm count at each point in the capillary tube, and the coloring represents the percent motility. Each point represents a technical replicate. 

**Supplementary Figure 9: Method development for cervical mucus penetration** To optimize the cervical mucus transit assay, several variants of the assay were performed with a single cervical mucus sample. In A) no sialidase was added as a control condition. In B), sialidase was added to sperm samples only. In C), sialidase was per-mixed with cervical mucus. Each point represents a technical replicate. Data in the top row are stacked graphs representing both sperm counts and contribution of each motility category divided by experimental condition, and the bottom row is split by motility category to compare different experimental conditions. 

B

A

**Supplementary Figure 10: Comparison of cervical mucin and bovine submaxillary mucin in sperm transit assay.** Cervical mucus transit assay was performed with 1.5% bovine submaxillary mucin in PBS, to compare sperm transit in the presence and absence of sialidase. Each point represents the average of three technical replicates, and error bars represent standard deviations.
